# Applications of Enzyme Technology to Enhance Transition to Plant Proteins: A Review

**DOI:** 10.3390/foods12132518

**Published:** 2023-06-28

**Authors:** Ourania Gouseti, Mads Emil Larsen, Ashwitha Amin, Serafim Bakalis, Iben Lykke Petersen, Rene Lametsch, Poul Erik Jensen

**Affiliations:** Department of Food Science, University of Copenhagen, 1958 Copenhagen, Denmark; mads.e.larsen@food.ku.dk (M.E.L.); ashwitha@food.ku.dk (A.A.); bakalis@food.ku.dk (S.B.); ilp@food.ku.dk (I.L.P.); rla@food.ku.dk (R.L.); peje@food.ku.dk (P.E.J.)

**Keywords:** protein functionality, plant-based foods, protein hydrolysis, cross-linking, deamidation, enzyme-assisted extraction, analogues

## Abstract

As the plant-based food market grows, demand for plant protein is also increasing. Proteins are a major component in foods and are key to developing desired structures and textures. Seed storage proteins are the main plant proteins in the human diet. They are abundant in, for example, legumes or defatted oilseeds, which makes them an excellent candidate to use in the development of novel plant-based foods. However, they often have low and inflexible functionalities, as in nature they are designed to remain densely packed and inert within cell walls until they are needed during germination. Enzymes are often used by the food industry, for example, in the production of cheese or beer, to modify ingredient properties. Although they currently have limited applications in plant proteins, interest in the area is exponentially increasing. The present review first considers the current state and potential of enzyme utilization related to plant proteins, including uses in protein extraction and post-extraction modifications. Then, relevant opportunities and challenges are critically discussed. The main challenges relate to the knowledge gap, the high cost of enzymes, and the complexity of plant proteins as substrates. The overall aim of this review is to increase awareness, highlight challenges, and explore ways to address them.

## 1. Introduction

### 1.1. Why Plant Proteins?

To date, food production has succeeded in offering safe, affordable food to billions of people around the world [1]. However, food practices are increasingly reliant on livestock, causing concerns about their effect on environmental and human health [2]. It has been estimated that food production is currently responsible for about 30% of the total anthropogenic greenhouse gas emissions [2], half of which are associated with animal-based, protein-rich foods [3]. This figure is projected to significantly rise by 2050 in a “business as usual” scenario [4], as a growing global population [5] and higher incomes are expected to prompt an increase in food and protein demand by up to 60% [6] and 100% [7], respectively. Moreover, as consumers’ high intake of animal proteins increases [8], it has been suggested that for heavy meat eaters, partial substitution of animal-based foods with plant-based foods may contribute to a healthy diet and reduce the risk of certain diseases such as type 2 diabetes, cancer, and bone diseases [9].

The plant-based food sector is thus expanding rapidly. It has been estimated that plant-based alternatives to animal-based foods can account for up to 10% of their respective global market shares in the next decade [10], although there are indications that this requires further efforts to provide consumers with healthy, palatable, and affordable analogues [11,12]. Proteins are at the center of this transition as they are not only essential nutrients but also an integral part of food structures and key in designing required food matrices such as gels, emulsions, and foams [13]. However, replacing animal proteins with plant proteins is not trivial, as the latter have different compositions, structures, and physicochemical properties from the former, and although significant achievements have been accomplished, a detailed understanding of their characteristics and how to transform them into desired foods is still lacking [1]. Often, plant proteins are said to have low functionality.

### 1.2. What Is Protein Functionality?

Protein functionality is a term often used with a degree of ambiguity to indicate characteristics of proteins that are relevant to their usefulness in certain applications, for example, foods. In 1981, Pour-El suggested the definition “any property, except its nutritional ones, that influences its utilisation”, which is widely accepted [14]. Examples of functional properties of proteins relevant to food applications are shown in Table 1. Of these, the ability of the protein to form and stabilize gels, emulsions, and foams is often considered key to developing the required food structures and textures. Methods to investigate protein functionality have recently been reviewed [15].

### 1.3. Seed Storage Proteins Are Major Candidates but Come with Challenges

Seeds are a major source of plant tissue harvested for human consumption [17]. Their protein content can reach up to 40% (dry matter) in certain legumes and oilseeds [17]. The majority of these proteins are storage proteins, probably the second most abundant protein group in plants after the leaf enzyme RuBisCO, a key enzyme for photosynthesis [18]. Seed storage proteins are therefore an important source of plant-based proteins. Due to their high protein content, legumes and defatted oilseed meal, a side stream of the oil industry currently used as feed [19,20], have high potential as protein sources.

Seed storage proteins are typically protein mixtures with exact compositions that depend on factors such as species, variety, and growth conditions. In nature, while animal proteins are readily available for use (e.g., milk used for growth, muscles for movement), storage proteins are typically designed to remain inert and store nitrogen and amino acids to be used for the initial development of the plant during germination [18,21]. They are produced in the endoplasmic reticulum, and in mature seeds, they are insoluble and densely packed in the cotyledon cells of the seed within membrane-bound vacuoles called protein bodies [22]. While this natural packing protects them from being unnecessarily used by the plant prior to germination, it presents challenges in their extraction and utilization by humans. Such challenges may refer to their inert nature, which limits their functional/structural properties, and their taste; for example, legume proteins are often referred to as “beany”, which is considered an off flavor. Modifications of plant proteins are often considered to improve their functional properties [23]. Today, analogues based on, or containing, plant proteins exist on supermarket shelves. However, they are generally less preferred by consumers compared to animal-based products for reasons such as their high price and reduced liking (e.g., due to suboptimal texture, taste) [24]. Additionally, they also typically require a high level of processing, which can substantially increase their environmental footprint [25].

### 1.4. Enzymes Can Assist in Promoting Plant Protein Utilization

Enzymes are protein molecules that can catalyze bioreactions. In the food industry, they have established uses in areas such as brewing, baking, cheese making, and juice clarification. As a result, the food-related enzyme market is constantly increasing, and currently accounts for about one third of global enzyme production [26].

Most of the enzymes used in food applications have similar roles industrially as they do in the living organisms from which they derive. For example, rennet is used by the stomach of calves to curd (i.e., hydrolyze and aggregate) milk proteins during digestion and by the industry to curd the same proteins for cheese production. As the plant-based market is increasing, potential enzyme applications in the field are also opening. However, existing knowledge from the dairy or other industries is in most cases not directly transferable to plant proteins due to their different, and often less researched, properties (see Section 4).

**Figure 1:**
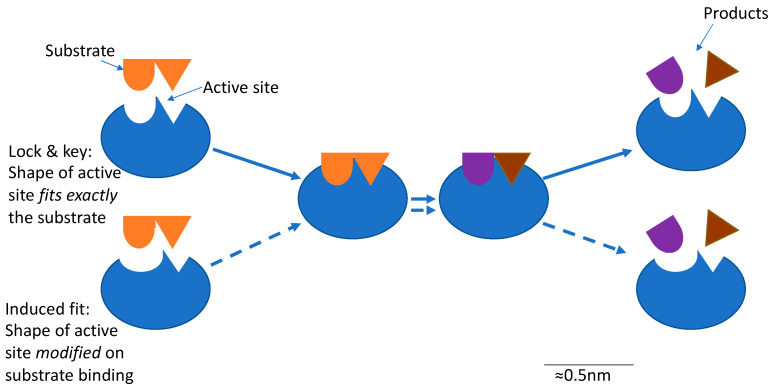
Schematic of the 2 models of enzyme action: Lock and key; induced fit.

Enzymes can modify the physicochemical and functional properties of plant proteins by catalyzing reactions such as hydrolysis, cross-linking, and deamidation. There are currently two widely accepted models for enzyme action, as indicated in Figure 1. In both models, the key to catalysis is a small region of the enzyme (about 10–20% of its volume) called the active site. According to the “lock and key” model, the shape of the active site fits exactly that of the substrate, while in the “induced fit” model, binding of the substrate induces structural changes to the active site for the reaction to happen. In all cases, binding of the enzyme’s active site with the region in the substrate molecule where the reaction occurs is required. The presentation of the protein molecule to the enzyme is therefore critical for the catalytic reaction. In the case of plant proteins, this can be challenging, as commercially available plant protein isolate ingredients are often sold as large aggregates that are difficult for enzymes to access (see Section 4.1).

Although research on enzyme uses for plant protein-based foods is growing, practical applications are still limited [18]. The challenges involved are those that are common to all enzyme applications, for example, their high cost, but also challenges specific to the task, for example, the typically large and inflexible structures of the plant proteins.

This review aims to present and discuss the current state of enzyme applications relevant to plant proteins, with a focus on seeds, with the desire to increase awareness, highlight challenges, and explore ways to address them. It is also the authors’ wish that this review could also be considered an educational tool, with the hope that it will serve as an instructive reference for students and anyone interested in the field.

## 2. Potential of Enzyme-Assisted Plant Protein Extraction

Plant proteins are extracted from seeds to produce protein-rich food ingredients known as concentrates (typically 40–80% purity) or isolates (typically >80% purity). Several methods can be used to extract the proteins, depending on the starting material and the desired properties of the final product. Isolates are typically produced by wet fractionation, which involves the initial milling of the seeds, the addition of solvents to extract the proteins, and the final spray drying of the product. This has enabled the production of high-purity plant proteins, which are currently used commercially to produce plant-based analogues. However, there are increasing concerns regarding its utilization. For example, it requires significant resources (e.g., water and energy) and generates significant side streams, which may result in an environmental impact that, on occasion, can be comparable to that of animal-based proteins [25]. In addition, the proteins can be subjected to extreme conditions (e.g., high pH, temperature >200 °C), which may affect their functional properties unfavorably, for example by prompting extensive denaturation and aggregation. As an alternative, dry fractionation has been suggested as a method that avoids the use of solvents and separates the dry milled seeds into protein-, starch-, and/or fiber-enriched fractions by techniques such as air classification or electrostatic separation. Although this may be a promising method for the future, utilization of the resulting protein-rich concentrates requires a better understanding of their properties. In addition, while wet fractionation may reduce or remove unwanted compounds, such as antinutritional factors, this is not the case for dry fractionation [27].

**Figure 2:**
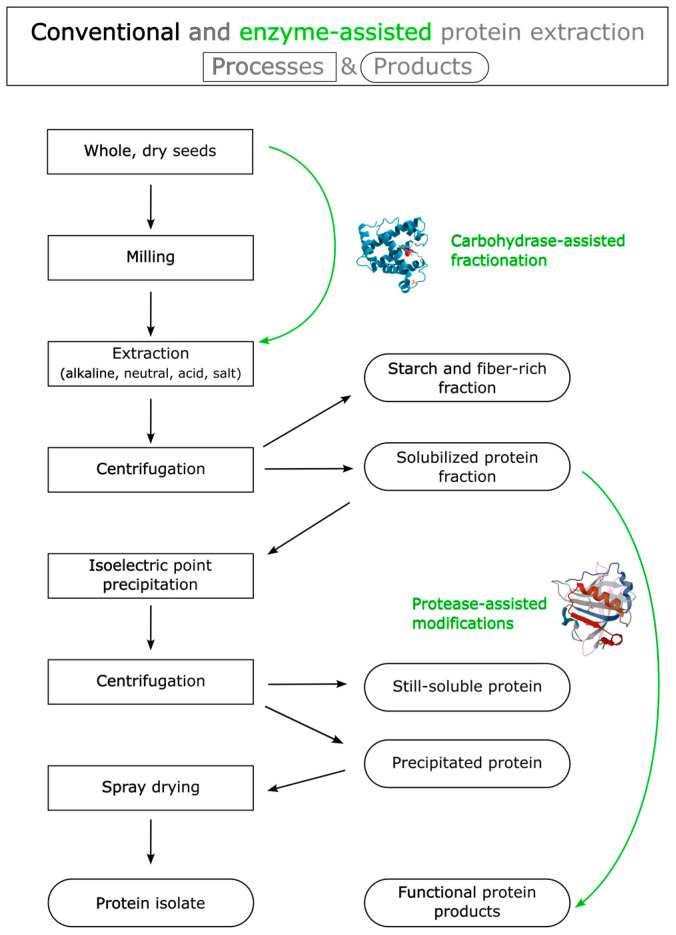
Schematic flowchart of enzyme-assisted protein extraction.

Enzymes may contribute to producing protein fractions and isolates with a reduced environmental footprint, e.g., by reducing the need for extensive milling or the use of solvents and improving the yield and functional properties of the acquired proteins [28]. Figure 2 shows an example schematic flowchart of how enzymes can be used to acquire functional proteins from seeds.

After removal of the husk and initial mechanical breakdown of the whole seed (milling), the main physical barrier to accessing the seed storage proteins is, depending on the milling conditions, the presence of the cell walls that surround the cotyledon cells [29]. Plant cell walls form a rigid protective layer around the cell that secures its structural integrity. They consist primarily of cellulose, hemicellulose, and pectin [30]. A degree of disruption of this sturdy layer is required to extract proteins, and this is currently achieved using physicochemical methods.

So-called cell wall-degrading enzymes are carbohydrases with specificity for degrading cell wall components (cellulose, hemicellulose, and pectin) [31]. They are naturally present in plants, where they play a key role in the ripening of fruits, releasing nutrients from specialized storage cells, or during germination [32,33]. Similar types of carbohydrases have been considered to aid in compromising the plant cell walls without the need for extensive physicochemical treatment [34]. In addition, proteases such as Alcalase^®^ and papain have shown potential to increase protein extraction yields by assisting in the separation of the plant proteins from their surrounding cellular matrix in the protein bodies [35]. It has been suggested that proteases can be more important than carbohydrases in extracting proteins from cereal bran and oilseed meals. However, it should be noted that the cell walls of these materials were already compromised during previous processing and oil extraction [35,36]. A different approach may therefore be required when extracting proteins from intact cells. An important consideration in enzyme-assisted protein extraction is the functional properties of the extracted material, which, depending on conditions, can be enhanced or reduced.

Enzyme-assisted extraction of plant proteins from a variety of sources has been explored, with seeds being the most prominent, but also side streams such as olive leaves and alternative food sources such as microalgae [37,38,39]. Example applications reported in the literature are shown in Table 2. Enzymes can assist in increasing the yield and may also influence the properties of the extracted proteins. Depending on the specificity and conditions, enzyme use during extraction has been reported to both improve and worsen the properties of the extracted proteins, as exemplified in Table 2.

## 3. Enzymatic Modifications to Improve Protein Functionality

Enzymes can modify the molecular characteristics of proteins, which can affect their functionality. There is, therefore, potential to produce proteins with tailor-made properties for use in specific food applications. The main pathways currently considered for enzymatic modifications are hydrolysis, cross-linking, and deamidation, and they will be presented separately in this section.

### 3.1. Plant Protein Hydrolysis

Using enzymatic hydrolysis to enhance protein functionality is old; for example, rennet has been produced industrially to cleave κ-casein in dairy applications since the mid 1800s [48]. However, interest is currently shifting to the less well understood plant proteins. The literature on the topic is exponentially increasing. Recently, a comprehensive review on functionalizing pulse proteins by enzymatic hydrolysis has been published [49], which we recommend to the interested reader. The present review briefly summarizes the potential mechanisms by which hydrolysis may affect protein functionality and introduces selected proteases relevant to food applications.

**Figure 3:**
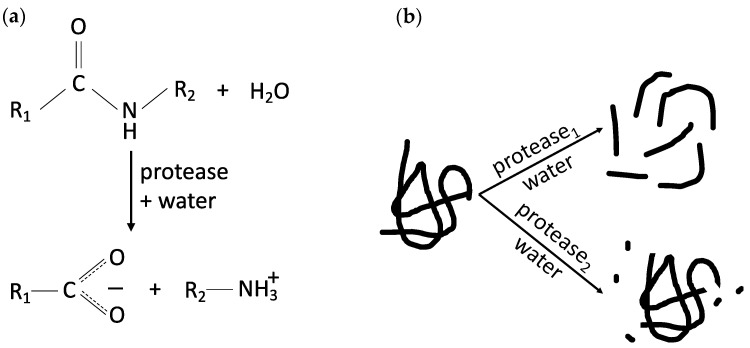
(**a**) Example of an enzymatic protein hydrolysis reaction showing cleavage of the peptide bond and production of two smaller peptides; (**b**) Simplified schematic of different scenarios for enzymatic protein hydrolysis. The top and bottom examples have similar degrees of hydrolysis (DH), but the functionality of the resulting hydrolysates differs. At the top, the protein is hydrolyzed into peptides of quasi-similar sizes; the interior of the protein is highly exposed. At the bottom, hydrolysis generated a few small peptides (or free amino acids), while the main part of the protein remained largely unaffected.

Protein hydrolysis refers to the breakage of peptide bonds and results in the formation of shorter peptides and single amino acids, depending on the type of protease used. An example of a reaction mechanism is diagrammatically shown in Figure 3a. The level of hydrolysis is often characterized by the degree of hydrolysis (DH), which is defined as the percentage of cleaved peptide bonds compared to the total peptide bonds available for cleavage in a protein hydrolysate [50]. The DH can be useful to quantify the extent of hydrolysis; however, it lacks information on the type of peptides (and/or amino acids) generated. This is exemplified in Figure 3b, which shows two potential scenarios of protein hydrolysis with similar DH but differing final composition and structure of the hydrolysates. In one scenario, hydrolysis results in peptides with comparable sizes, where the interior of the “parent” protein is highly exposed; in the other, the resulting mixture contains small peptides while most of the “parent” protein remains largely untouched. The properties of the two hydrolysates are expected to be different. DH should therefore be used with caution and an understanding of its limitations.

A 10% DH threshold has been suggested to distinguish “extensive” (DH ≥ 10%) from “limited” (DH < 10%) hydrolysis. Although this limit serves as a useful guideline, it has been somehow arbitrarily chosen and does not necessarily relate to any functionality threshold of the resulting hydrolysates [51]. The exact DH of an enzymatic hydrolysis depends on factors, including the protein that is being hydrolyzed, the concentration and specificity of the protease(s), and the reaction conditions [52].

Partial enzymatic hydrolysis is one of the most investigated techniques to modify the functional and nutritional properties of plant proteins. During protein hydrolysis, peptide bonds are cleaved. As a direct consequence, the number of carboxyl and amino terminals increases, and thereby the number of ionizable groups increases. Another possible effect of hydrolysis is protein unfolding and exposure of the interior of the “parent” protein molecule to the solution (as in the top scenario in Figure 3b). As this interior is often high in hydrophobic amino acids, surface hydrophobicity and the associated hydrophobic interactions between or within peptides may also be enhanced. Studies have also shown that protein hydrolysis using specific proteases may further modify the sensory profile (taste or texture) of the resulting plant-based foods, increase their digestibility, and/or reduce possible allergenicity [53,54].

High levels of hydrolysis can have negative effects on the protein’s functional properties. As an example, excessive proteolysis may result in small peptides and amino acids being unable to form emulsions, foams, and gels, or it may release bitter peptides [55]. Examples from the literature on the mechanisms by which hydrolysis may affect the food-related functionalities of plant proteins are shown in Table 3. This table shows some trends, but it should be treated with caution before generalized conclusions can be drawn, as the systems are often highly sample-specific.

A range of proteases derived from different sources, including animals, plants, microbes, and fungi, are currently commercially available for hydrolysis [52]. The origin of the enzymes should be considered in the production of special diets such as vegetarian or vegan diets, as some sources may not be compatible with all diets. Some proteases have broad specificity and can cleave almost any peptide bond, while others have more narrow selectivity for substrates. When comparing animal- to plant-derived proteases, the former usually have greater specificity compared to the latter [52]. Proteases can also be endo- or exo-active based on whether they cleave in the middle or near the end of the polypeptide chain, respectively. The major sources and activities of proteases with potential in the food area are presented in Table 4.

### 3.2. Cross-Linking

Protein cross-linking results in the formation of covalent (isopeptide) bonds between the polypeptide chains within the same molecule (intramolecular) or between two different molecules (intermolecular) [106]. Transferases, hydrolases, and oxidoreductases have been shown to possess protein cross-linking enzymatic activity [107,108]. In food applications, the most frequently encountered cross-linking enzyme is the transferase transglutaminase (TG), followed by the oxidoreductases tyrosinase, laccase, and peroxidase [108].

**Figure 4:**
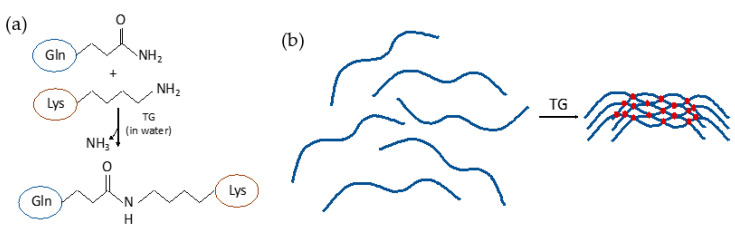
(**a**) Enzymatic protein cross-linking reaction with transglutaminase (TG) showing the resulting isopeptide covalent bond; (**b**) simplified schematic of an example protein network induced by TG; the enzymatically generated covalent bonds between peptides are shown in red dots (adapted from [105]).

TG catalyzes the formation of glutamyl-lysyl isopeptide bonds between γ-carboxamide groups of glutamine (E) residues and ε-amino groups of lysine (K) residues in primary amines, peptides, and proteins (Figure 4a) [109]. It is a common enzyme in nature, involved in processes such as blood coagulation in mammals, plant growth, or spore coat formation in microbes. While initially sourced from mammals, such as guinea pig liver, at present microbial TG is preferred due to the lower production cost and animal welfare concerns [109,110]. In addition, contrary to mammal TG, microbial TG does not require calcium as a cofactor, making its use more versatile.

By cross-linking proteins, TG can modify their functional properties. Under favorable conditions, it can boost protein network formation, as schematically shown in Figure 4b. Due to its previous widespread use in the meat industry to “glue” meat pieces together, TG was commonly referred to as “meat glue”, but this name is now often avoided due to health-related concerns about the resulting “glued” meat. Extensive consumption of TG has been associated with adverse health effects such as increased risk for certain autoimmune and neurodegenerative diseases [111,112]. However, it may also have beneficial health effects, such as increasing the sense of satiety and reducing the allergenicity of foods [111]. TG is a food-grade enzyme with high potential for producing satisfactory food products if used within the recommended guidelines.

Examples of how TG may affect the properties of protein-based food matrices are shown in Table 5. The effect of TG treatment has been shown to depend, among other things, on the exact protein and amino acid content of the substrate. For example, higher glycinin content was shown to increase the porosity and stiffness while reducing the water-holding capacity of soy protein-based gels [113]. While TG is typically associated with gelled materials, where it has been reported to increase the strength and firmness of gels, other properties, such as the insulating effect of edible films for food packaging applications, have also been investigated, as shown in Table 5.

### 3.3. Deamidation

**Figure 5:**
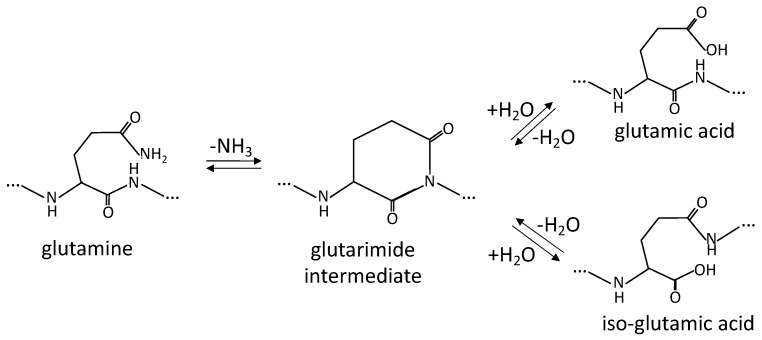
Diagrammatic deamidation reaction for glutamine, showing the glutarimide intermediate and the products glutamic acid (α-Glu) and iso-glutamic acid (γ-Glu) (adapted from [124]).

Deamidation refers to the hydrolysis of the amide linkage in the side chains of asparagine (N) and glutamine (Q) residues to form their corresponding carboxylic acid derivatives and ammonia [122,123], as schematically exemplified for glutamine (Q) in Figure 5.

Popular enzymes for protein deamidation include peptide-glutaminase and protein glutaminase (PG). As their names indicate, the former is active on short peptides, while the latter can deamidate larger peptides or proteins [125]. PG was first isolated from a bacterium in 2000 and has since gained popularity due to its targeted specificity. Proteases (e.g., trypsin, chymotrypsin, and pronase) and TG have also shown potential for deamidation [126], although their utilization should be implemented with consideration of their other actions (i.e., protein hydrolysis and cross-linking, respectively) on the proteins. A summary with examples of how deamidation may affect protein functionality is shown in Table 6.

## 4. Challenges and Opportunities

Research to date suggests that enzymes offer a promising “shortcut” to accelerate the transition to a sustainable, plant-based future. They can assist in the gentle extraction of functional plant proteins, for example, by compromising the plant cell wall and releasing proteins from their protein-fiber network, or they can modify plant protein functionality post-extraction to match the required functional properties. They offer a potential sustainable option that can further contribute to the “greener” label of the resulting foods, as they may reduce the need for additives. However, exploiting enzymes to their full potential presents a range of challenges and opportunities that need to be considered. Some of these challenges are generic to a range of substrates, while others are specific to plant proteins. This section presents a selection of challenges.

### 4.1. Plant Proteins as Substrates: Large, Aggregated, Variable Mixtures

As previously mentioned, enzymatic reactions require the binding of the substrate to the enzyme’s active site. Active sites can be positioned on/near the enzyme’s surface, or they may be deeply buried, for example, in hydrophobic pockets, which limits accessibility and can make the enzyme more specific. The accessibility of the substrate is equally important. In the cases of small or otherwise accessible molecules, for example, the disaccharide lactose, the linear cellulose, or the loosely packed gelatinized starch, substrate accessibility is typically straightforward.

**Figure 6:**
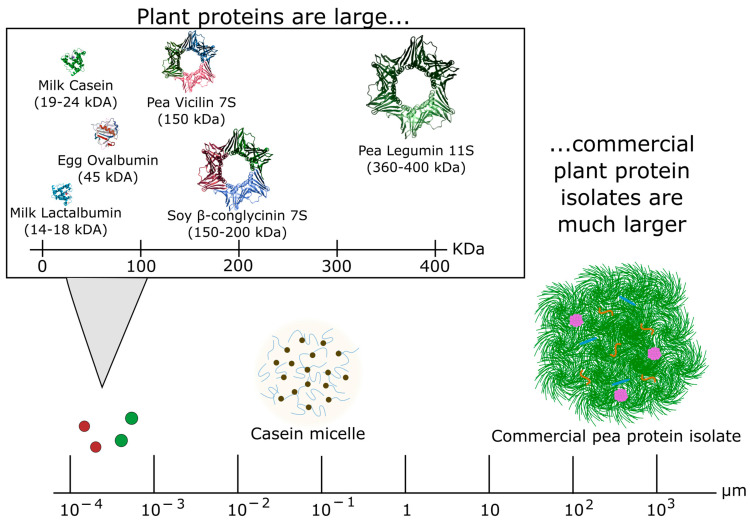
Schematic showing the relative sizes of milk/egg proteins, plant proteins, casein micelles, and commercial plant protein isolates, with images adapted from [135,136,137,138,139,140,141,142,143,144]. Note that 7S proteins are trimers whereas the 11S proteins are hexamers, comprising two subunits. In the figure, the pea legumin 11S shows one of the two subunits. In the size line, the red and green dots show approximate sizes of the animal and plant proteins, respectively.

However, plant proteins can be challenging substrates. One reason for that is their large molecular size, compared to other proteins such as dairy or egg ovalbumin (see Figure 6), and often compact, globular structure, which reduces accessibility to their interior. In addition, during the production of protein-rich fractions such as commercial protein isolate ingredients, the proteins are subjected to pH and temperature conditions that can cause denaturation and aggregation of the proteins. As a result, they can form large aggregates of the order of 100 μm (see Figure 6), which severely restricts the accessibility of proteins found at the inner part of the particles. It is noted that large structures, for example, casein micelles with average sizes of about 150 nm, can also be relatively accessible substrates if the required enzymatic reaction takes place at the surface of the particle, as it happens during cheese production. However, this is not the case with plant proteins. As a result, it is possible that hydrolysis may release small peptides or single amino acids from the surface of the particle, while the bulk could remain largely untouched. This is one of the reasons why it can be difficult to extrapolate existing knowledge of enzyme use, for example, from dairy applications to plant–protein substrates.

Another challenge associated with plant proteins as substrates refers to their diversity, as they are typically mixtures of different proteins, as well as their variability. In addition to the protein source and growth conditions, an important source of variability originates from the extraction and drying that the proteins undergo during the production of the isolates. These may affect the physical characteristics of the proteins, such as the level of aggregation, but also the chemical properties and composition of the material. As a result, the properties of plant protein isolates may vary considerably depending on the supplier and/or batch.

Predicting the outcomes of enzymatic modifications can be challenging, and it has been shown to depend on factors such as the specific substrate [145], enzyme [146], and conditions such as enzyme concentration [147]. For example, soy protein isolate treated with Flavourzyme^®^ showed increased functionality compared to chickpea protein treated under similar conditions [145]; pea protein isolate treated with trypsin showed higher solubility compared to the same protein treated with a range of other proteases [146]; and oat protein hydrolyzed with Alcalase^®^ was found to be more functional at an enzyme concentration of 6% compared to lower or higher concentrations [147]. Developing proteins with tailor-made functionality may therefore become protein-specific, supplier-specific, and batch-specific. This needs to be simplified to achieve a meaningful understanding of enzymatic modifications.

### 4.2. Plant Proteins May Contain Protease Inhibitors

Protease inhibitors are small proteins that can inhibit the action of digestive proteases, typically by binding to the target enzyme and thus restricting accessibility to the active site [148]. They can be found in high concentrations, up to 10% of the total protein content, in storage tissues but are also detectable in leaves [148]. Their role is to defend the plant from herbivores and pests by making the plant antinutritious. In humans, although they have been linked with certain potential therapeutic activities [149], they are generally considered unwanted in large amounts as they may reduce protein digestion and absorption. Being proteins themselves, they are generally susceptible to high temperatures [150], therefore they are often inactivated during cooking. However, they may have a role during protein extraction, which is carried out at room temperature, during enzymatic modifications of unheated proteins, or when heating only partially deactivates them.

### 4.3. Understanding Substrate Presentation in Complex Systems

Research to date on enzymatic reactions has typically been carried out in solutions or dilute suspensions. However, food production may require enzymatic reactions in complex systems where the presentation of the enzyme and accessibility to the substrate can become challenging. To date, knowledge of substrate presentation in complex food structures is still limited. This includes substrates incorporated in concentrated mixtures, where there is limited water and therefore restricted mobility to support the reaction; substrates in multi-component systems, where protein interactions with other components alter its molecular structure; or substrates in previously set systems such as gels, where accessibility is again restricted and dependent on how the enzyme can diffuse through the gel network. This has been previously identified for emulsions and gel networks involving dairy proteins, while much less is known for plant-based proteins [151,152,153]. Research in this field is expected to increase in the future [154].

### 4.4. Enzyme Inactivation

When using enzymes in food production, an additional processing step that may need to be considered is their inactivation. Several inactivation methods exist, with the most popular being heating the material to temperatures where the enzymes are denatured and therefore lose their activity. However, in addition to enzyme inactivation, heating will affect material properties and need to be well controlled. This has been observed for dairy proteins [155]. In addition, there may be occasions, for example, after protein gelation to produce a yogurt or cheese-like food, where heating may be undesirable and another option should be considered. In yogurts, pH reduction through fermentation may help with enzyme inactivation. An open, probably application-specific, question remains whether enzyme inactivation is always necessary or if it could be avoided without compromising food quality. For example, reduced molecular motility in gelled systems is expected to limit substrate-enzyme collisions for kinetic reasons; therefore, a gradually gelling system may result in gradually reduced enzymatic activity. In addition, enzymes lose activity over time, which why they are stored at reduced temperatures. It may therefore be possible to bypass enzyme inactivation by controlling dosage and processing/storage conditions; however, this requires further research into each enzyme’s kinetics and characteristics.

### 4.5. From Enzymatic Reactions to Food Products

**Figure 7:**
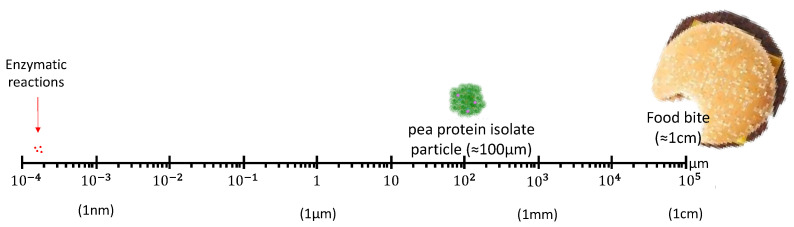
Relative sizes from molecules (proteins) in foods. Enzymatic reactions at a molecular level affect the properties of the food bite (cm in size).

Although significant progress has been achieved, there is still a gap in understanding how to link what happens at a molecular level with the final properties of a food product. Enzymatic reactions are no exception. They can alter the molecular features of proteins, yet foods have characteristic structures at micro and millimeter scales (see Figure 7), which highly determine their properties and consumer response. It is therefore important to understand how enzymatic modifications of plant proteins affect their interactions with other ingredients and how these interactions can be exploited to build higher-order desirable, predictable structures during processing.

The key to promoting plant-based proteins in foods is therefore to identify characteristics (e.g., size distribution, surface hydrophobicity, amino acid composition) that are important in determining the interactions of the proteins with other compounds in the food matrix during processing. The challenge of linking length scales has been reported for products such as cheese [156]. A detailed understanding of the link between the biochemical properties of the proteins, processing conditions, and food material properties is currently incomplete and will enable accurate prediction of enzymes and conditions to optimize plant protein utilization.

### 4.6. Optimizing Reaction Conditions

The extent and kinetics of enzymatic reactions depend on conditions including, but not limited to, temperature, pH, reaction time, enzyme, and substrate concentration [157]. Optimizing these factors may therefore contribute to gaining the desired enzymatic treatment at the lowest cost, and relatively simple optimization assays could, on certain occasions, be used [158]. However, the process is complicated by the fact that enzyme cocktails, containing enzymes with varying optimal working conditions, are often used [28]. In addition, plant proteins make complex substrates of mixed, aggregated proteins, as discussed in Section 4.1. Optimizing reaction conditions with plant proteins therefore requires knowledge of exact enzyme and substrate properties. Optimal conditions may sometimes vary with time; for example, it may be beneficial to slowly heat up or acidify the reaction mixture. Process optimization may therefore be required to identify appropriate conditions for the enzymatic reactions.

### 4.7. Challenges on Scaling up Enzymes

Biological processes present significant challenges when operated at manufacturing scale [159]. There is limited literature on the challenges relevant to industrial use of enzymes for plant-based foods; rather, the information comes more from the use of enzymes for biofuels and general biocatalysts. Techno-economic considerations can be a limiting factor in the implementation of biocatalysis [159].

For processes such as protein hydrolysis to be financially viable at industrial scales, a high solids content has to be used, e.g., >15%. This imposes significant mass transfer limitations and challenges in predicting and operating industrially [160]. Mass transfer of enzymatic hydrolysis in a human digestion context has been found to depend on flow parameters (e.g., laminar/turbulent), the properties of the material (e.g., viscosity), and mixing [161,162]. Efficient reactor designs, i.e., process intensification technologies, have been proposed for increasing mass transfer and doubling reactor performance [163]. For example, for enzymatic biodiesel production, ultrasound and microreactor technologies can improve mass transfer and could be scalable [164]. There is limited data and methodologies on cost estimation and uncertainty, which currently limits the application of technologies at an industrial scale. Bioprocesses in general tend to suffer from much higher intrinsic variability compared to chemical processes. There is an overall need to understand robustness on an industrial scale. This requires an understanding of process corridors, understanding how variances propagate not only across individual unit operations but also across process lines, and understanding the relationship between input variables and outputs. In this direction, sensors and data-driven approaches can work to enable robust processes [165].

Scaling up the enzymatic hydrolysis of worm protein has been investigated [166]. The authors used two model reactors and identified four key dimensionless numbers. The developed models accurately predicted rates of hydrolysis. The performance of enzymatic systems over long timescales can also be a challenge. The application and scale-up of enzymes for the generation of lactulose have been investigated [167], indicating that it is possible for some enzyme mixtures to operate for a long enough time to ensure that they can be used at an industrial scale.

Overall, despite challenges, enzyme technologies have found applications at industrial scales, and the plant-based food sector could benefit from established knowledge from other fields, including general biocatalysis and biofuels.

### 4.8. Synergies with Other Techniques

In food production, enzymes would be part of a series of processes, and potential synergies could therefore be exploited. For example, whether proteins are heated before or after enzymatic treatment may affect substrate accessibility and can therefore be important in determining enzymatic reactions and the properties of the resulting material. Combinations that involve novel technologies may also be considered. For example, enzyme-assisted supercritical fluid protein extraction and ultrasound-assisted enzymatic extraction have shown potential for improving yield and protein quality [34].

### 4.9. Choosing Solvents

Enzymatic reactions require a solvent, and to date, aqueous reaction media have been commonly used industrially. Around the 1980s, non-aqueous enzymology was introduced. Initially, it involved water-miscible organic solvents, such as ethanol, but technological advancements opened a range of other opportunities. Examples include the use of biphasic mixtures where the enzyme is emulsified in water-immiscible solvents, the use of reversed micelles to stabilize enzymes in water/organic mixtures, or suspensions of freeze-dried enzyme powders in anhydrous organic solvents or supercritical fluids [168]. Nonaqueous solvents are particularly useful when the compounds involved in the reaction have poor solubility in water, such as in lipase-catalyzed reactions, and they have advantages such as easy recovery of the non-soluble enzymes and reduced potential for bacterial contamination [168]. In food applications, solvents regarded as hazardous, such as n-hexane and chloroform, are considered undesirable [169].

Ionic liquids have recently gained attention as potential green solvents for enzymatic reactions. Ionic liquids are fluids containing large, bulky ions with a melting temperature below 100 °C [170]. Ionic liquids interact with both polar and nonpolar compounds, making them useful in the extraction of a range of different compounds. Proteins extracted using ionic liquids as solvent often maintain their native conformation and, thereby, functional properties to a higher degree than if other solvents are used. Additionally, their high viscosity makes enzymes more resilient to higher temperatures [157]. This means costs associated with enzyme use, a major barrier in their industrial application, can be lowered by using ionic liquids as solvents.

### 4.10. Addressing the Challenge of Costly Enzymes

One of the largest hindrances to industrial enzyme use is their high cost. As mentioned earlier, the use of certain solvents or reaction media may reduce operating costs by providing resilience and prolonging the lifetime of the enzymes. Enzyme immobilization has also been considered to reduce the cost of enzymatic reactions, as it may increase efficiency and facilitate the recovery of the enzyme [171].

**Figure 8:**
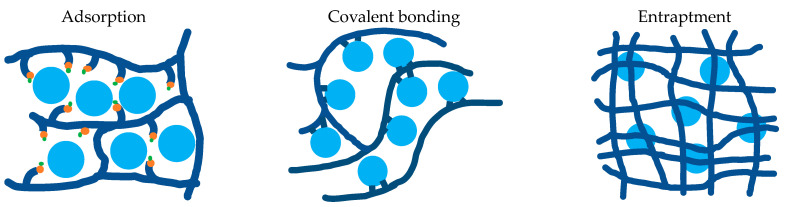
Schematic drawing of the three main methods of enzyme immobilization (adapted from [172]).

There are three main methods of immobilization: adsorption, covalent bonding, and entrapment (see Figure 8). Physical adsorption is the oldest method, with origins at the beginning of the 20th century. It involves the binding of the enzyme with the absorbent (e.g., collagen, silica gel, or glass) by non-covalent interactions [172]. Covalent immobilization (e.g., on cellulose) was introduced in the mid-1900s, and while it is typically more tedious than adsorption, it can result in firmer binding of enzymes to their support. Entrapment refers to the immobilization of enzymes within a solid or semi-solid matrix, such as a gel [173]. Entrapped enzymes are not attached to any compound, and therefore steric issues, for example, binding of the enzyme in a way that hides its active site, are overcome. The manufacture of champagne by Moët & Chandon uses this immobilization method [172].

## 5. Conclusions

Enzymes have proven to be a great resource in a variety of industrial applications, from biofuels to chemical synthesis and washing powder. Enzymes have also been used to a large extent in the food industry, especially in the dairy industry. As the food industry shifts to the production of plant-based foods with a focus on variation, palatability, and functional properties to replace animal products, enzymes seem like an obvious choice of tool to explore. In addition to protein extraction, examples of applications covered in this review in which enzyme use is being explored are partial protein hydrolysis, cross-linking, and deamidation. Knowledge on how enzymes may affect plant protein extraction and functionality and how this can be used in new product or process development is still largely empirical. The emergence of commercially available enzyme mixtures that promise plant-based analogues with enhanced properties, such as taste, by modifying the proteins during or post-extraction shows an increasing industrial interest and a trend in expanding enzyme uses for the production of the next generation of plant-based foods.

Enzymes can modify the structural properties of proteins. By reducing molecular size, partial hydrolysis has overall shown the potential to increase functionality in terms of solubility as well as emulsifying, foaming, and gelling properties. However, the actual effect has been reported to be highly substrate and enzyme specific, while it also depends on the conditions; for example, small peptides and amino acids resulting from extensive hydrolysis have been shown to possess limited structuring properties, such as emulsifying activity. Cross-linking produces covalent bonds between protein residues, and its effect is often associated with increased strength and firmness of protein-based gel networks in a manner that is, up to a certain degree, dose-response-related. Deamidation increases the charge of protein molecules and has shown the potential to increase the solubility and structuring properties of proteins.

Other attributes of enzymatic modifications of plant proteins include the potential to aid in the removal of antinutritional compounds such as allergens and protease inhibitors and to change the taste of the proteins. For example, hydrolysis has been reported to potentially increase the bitterness of the proteins, while deamidation has shown potential to increase the umami taste of the material.

This review has also shed light on the challenges that need to be addressed in the application of enzymes to plant products. These included highly variable and inaccessible protein structures and the balancing of extraction yield versus the quality of the extracted protein. The authors of this paper are confident that research in the area will continue to increase in parallel to the increased utilization of enzymes in the production of satisfactory plant-based food alternatives in the coming years.

## Figures and Tables

**Table 1:** Examples of functional properties of proteins relevant to food applications (adapted from Kinsella 1979, as presented by [16]). Properties have been grouped to showcase the various aspects of protein functionality.

Property Category	Example Functional Properties
Sensory	Color; flavor; smoothness; grittiness; mouthfeel
Hydration	Solubility; dispersibility; swelling; wettability; water absorption; water holding capacity; protein–water interactions
Surface properties	Emulsification; foaming; lipid-binding; surface hydrophobicity; amphiphilicity; surface charge; contact angle; oil holding capacity; film formation
Texture-related properties	Viscosity; elasticity; gelation; aggregation; extrudability; emulsification and foaming; adhesion; stickiness; chewiness; viscoelasticity; fiber formation ability

**Table 2:** Example uses of enzymes in protein extraction across protein sources. It is noted that Pectinex^®^ and Alcalase^®^ are commercial preparations of enzymes that degrade mainly pectin and proteins, respectively; Celluclast^®^ and Viscozyme^®^ degrade cell walls; Depol^®^ and Shearzyme^®^ degrade predominately xylan.

Protein Source	Enzyme (s)	Extraction Conditions	Yield	Quality	References
Defatted soybean cake	Cellulase, xylanase, pectinase	Mildly alkaline	45%	Near-native; higher solubility and emulsifying properties when using enzymes in extraction	[40]
Pea	Papain	Mildly alkaline	58%	Small peptides, amino acids; extensive proteolysis reduced emulsifying properties (also reported in Section 3.1)	[41]
Chickpea	Arabinofuranosidase or cocktail of cellulase and xylanase	Alkaline	93%	Increased yield and functional properties of the protein isolate with both enzymatic treatments compared to alkaline extraction alone.	[42]
Rapeseed cake	Pectinex^®^, Depol^®^, Celluclast^®^	Neutral	Up to 74%	Not reported	[43]
Lentil	Viscozyme^®^	Mildly acidic for the enzymatic pre-treatment, then alkaline	62%	Similar yield but higher purity and improved functional properties when using enzymes, compared to alkaline extraction alone.	[44]
Sesame	Neutrase^®^, Pectinex^®^	Not reported	90%	Small peptides; extensive use of carbohydrases reduced purity as the product contained solubilised carbohydrates.	[45]
Akebia trifoliata	Cellulase	Alkaline	20%	Higher purity and functional properties compared to alkaline extraction alone	[46]
Red seaweed	Alcalase^®^, Celluclast^®^, Shearzyme^®^	Mildly alkaline	90%	Large, highly functional peptides under investigated conditions, despite using proteases	[47]

**Table 3:** Mechanisms of how hydrolysis may affect the functionality of plant proteins.

Property	Mechanisms through Which Hydrolysis May Increase It	Mechanisms through Which Hydrolysis May Reduce It	Examples (with References)
**Solubility**	Size reduction. Increase of ionizable groups.	Hydrophobic interactions of newly exposed groups.	Results vary considerably, but enzymatic hydrolysis appeared to increase solubility of chickpea [56], peanut [57], sunflower [58], oat [59], rice endosperm [60], and pea [55] protein at DH up to 23%.
**Surface Hydrophobicity**	Exposure of hidden hydrophobic groups to the surface.	Hydrophobic interactions of newly exposed groups, particularly at high DH.	Effect heavily depends on enzyme specificity and conditions. Can increase surface hydrophobicity of soy protein isolate [61]; hemp protein isolate [62]; brewers spent grain protein concentrate [63].
**Emulsification**	Increased solubility. Increased surface hydrophobicity. Exposure of hidden hydrophobic groups that can adhere to the O/W interface. Increased amphiphilicity. Increased molecular flexibility and possibly disruption of the compact molecular structure [64,65].	Extensive reduction in molecular size and hydrodynamic diameter at high DH. This may reduce potential of interfacial interactions and viscoelasticity of the resulting film. Reduced surface hydrophobicity at high DH.	Depending on conditions, limited hydrolysis (generally about up to 2–3% DH) overall increased emulsion capacity and stability in protein-stabilized O/W emulsions with rice bran albumin and globulin [64]; potato protein concentrate [65]; pea protein isolate [55]; chickpea protein isolate [66].
**Foaming**	Similar to emulsification, factors that enhance surface interactions of the hydrolysates increase foamability.	Similar to emulsification, factors that decrease surface interactions of the hydrolysates reduce foamability.	Largely depending on conditions. At low DH, foaming properties increased for soy protein [67], sunflower protein isolate [68], pea protein isolate [55,69].
**Gelation**	Factors that enhance protein–protein and reduce protein–water interactions. “Loosening” of the compact protein molecules.	Factors that enhance protein-water and reduce protein-protein interactions. Reduced molecular size. Reduced hydrophobicity. Increased surface charge.	Limited hydrolysis increased gelling properties of soybean proteins [70]; pea proteins [71]; peanut protein isolate [57]; oat protein [72]; rice endosperm protein [73]; defatted soy flour [74]; sunflower protein [75].

**Table 4:** Main sources and activity of proteases with potential in plant protein-based food applications (note that some of the commercial enzymes are mixtures). Details, including stereospecificity of the enzymes, are out of the scope of this review and are therefore omitted.

Enzyme	Major Sources	Action Site	Product	References
Trypsin	Porcine or bovine intestine	Highly specific. Cleaves C-terminal to arginine (R) and lysine (K) residues. Less effective if acidic residue (glutamate (E) or aspartate (D)) is near the cleavage site. May cleave before proline (P).	Small peptides	[76,77,78]
Pepsin	Porcine gastric mucosa	Broad specificity, with overall preference to cleave after bulky aromatic residues (maybe favoring phenylalanine (F)), leucine (L), and possibly methionine (M). Cleavage after histidine (H), lysine (K), arginine (R), proline (P) usually not as favored, unless adjacent to residues such as leucine (L) or phenylalanine (F).	Small peptides	[79,80,81]
Carboxy-peptidase (CP)	CP-A from bovine pancreas; CP-B from bovine or porcine pancreas; CP-Y (yeast CP) from baker’s yeast.	CPs are exopeptidases that cleave the carboxy end of proteins and peptides, usually one residue at a time. Depending on their substrate preference they can be classified as CPs-A (prefer aromatic and large aliphatic sidechains, hydrolyze slowly glycine (G) and acidic residues, rarely proline (P) and basic residues); CPs-B (with narrower specificity than CPs-A and preference towards the basic residues arginine (R), lysine (K) and some action on neutral amino acids); and CPs-C (can release proline (P) and other amino acids). CP-Y has broad specificity, similar to CP-A but cleaves rapidly glycine (G) and leucine (L), and slowly phenylalanine (F).	Typically single amino acids	[82,83,84,85]
Amino-peptidase (AP)	Microbes and porcine kidney.	APs are exopeptidases that cleave the amino end of proteins and peptides. They can be classified to aminoacylpeptidases, dipeptidyl- and tripeptidyl- peptidases (i.e., releasing single amino acids, dipeptides, tripeptides, respectively), with a tetra-peptidase recently reported. If acting only on di- or tri- peptides, they are di- and tri-peptidases, respectively. Based on substrate specificity they are classified into 2 categories: broad and narrow.	Amino acids, di-peptides, tri-peptides, rarely tetra-peptides	[86,87]
Alcalase	Microbes	Has broad specificity. Reported to cleave bonds on the carboxyl side of glutamic acid (E), methionine (M), leucine (L), tyrosine (Y), lysine (K), and glutamine (Q), also at phenylalanine (F), tryptophan (W), alanine (A), serine (S).	Small peptides	[88,89,90,91]
Plasmin or fibrinolysin	From bovine plasma or microbes	Has similar specificity to trypsin, but less efficient. Cleaves after arginine (R) and lysine (K) residues.	Small peptides	[92,93,94]
Flavor-zyme^®^	Microbial (*Aspergillus oryzae*)	Broad specificity, mostly endo activity	Small peptides and amino acids	[95,96]
Protamex	Microbial (*Bacillus*sp.)	Broad specificity.	Small peptides	[95,97]
Neutrase^®^	Microbial (*Bacillus amyloliquefaciens*)	Broad specificity	Small peptides	[95]
Corolase 7089	Fungal neutral protease	Broad specificity	Small peptides	[97]
Pronase	Microbial (*Streptomyces griseus*)	Broad specificity	Amino acids and peptides	[98]
Prolidase	Microbial	Cleaves before proline (P) or hydroxylproline in dipeptides.	Amino acids	[99]
Ficin	Fig (*Ficus carica*)	Generally prefers to cleave after aromatic residues e.g., tyrosine (Y), phenylalanine (F); exact specificity depends on form.	Small peptides	[100,101,102]
Papain	Papaya (*Carica papaya*L.)	Has broad specificity, with reported preference to cleave bonds at arginine (R), lysine (K), and phenylalanine (F).	Small peptides	[52,88,103,104]
Bromelain	Fruit or stem of pineapple (*Ananas comosus*L.)	Broad specificity.	Small peptides	[88,100,105]

**Table 5:** Examples of how transglutaminase-mediated cross-linking may affect protein functionality.

Observation	Protein Source (with Ref)
Increased gel strength and firmness; Some studies mention increased water holding capacity.	Faba bean protein isolate [114]; pea protein isolate [115]; soy protein [116]; soybean milk [117,118]
Decreased solubility and increased surface hydrophobicity	Peanut protein isolate [119]; vicilin-rich kidney protein isolate [120]
Reduced CO_2_ and O_2_ permeability in edible protein films (for food packaging applications)	Bitter vetch protein films [121]

**Table 6:** Examples of how deamidation may affect protein functionality.

Observation	Possible Mechanism	Protein Source (with References)
Increased solubility and emulsifying/foaming properties, particularly at neutral pH	Increase in protein charge and associated inter-molecular repulsions may increase solubility; higher solubility may increase foaming and emulsifying properties.	Wheat gluten [127]; soy [128]; oat [129]; coconut [130].
Improved taste, for example through decreasing binding affinity of proteins to tastants (vanillin), which become free, or by decreasing bitter taste and enhancing umami	By reducing binding affinity of proteins to tastants, therefore increasing the “free” tastant concentration.	Coconut [131,132]; soy [131,132]; wheat gluten [133,134]; wheat gluten hydrolysates [133,134].
Reduced allergenic potential	Conformational changes of the proteins, particularly for proteins high in glutamine residues that are susceptible to deamidation	Wheat gluten [127]

## Data Availability

Not applicable.
